# Prolonged Running Using Bionic Footwear Influences Lower Limb Biomechanics

**DOI:** 10.3390/healthcare9020236

**Published:** 2021-02-23

**Authors:** Xinyan Jiang, Xiaoyi Yang, Huiyu Zhou, Julien S. Baker, Yaodong Gu

**Affiliations:** 1 Faculty of Sports Science, Ningbo University, Ningbo 315211, China; jiangxinyan168@163.com (X.J.); yangxiaoyiyiyi@126.com (X.Y.); 2 Research Academy of Grand Health, Ningbo University, Ningbo 315211, China; zhouhuiyu@aliyun.com; 3 School of Health and Life Sciences, University of the West of Scotland, Scotland G72 0LH, UK; 4 Centre for Health and Exercise Science Research, Department of Sport, Physical Education and Health Hong Kong Baptist University, Hong Kong 999077, China

**Keywords:** footwear, prolonged running, bionic science, running biomechanics

## Abstract

The running biomechanics of unstable shoes have been well investigated, however, little is known about how traditional neutral shoes in combination with unstable design elements and scientifically (bionic) designed shoes influence prolonged running biomechanics. The purpose of this study was to investigate biomechanical changes for a typical 5 km run and how footwear technology may affect outcomes. Sixteen healthy male recreational heel strike runners participated in this study, and completed two prolonged running sessions (neutral shoe session and bionic shoe session), with 7 to 10 days interval between sessions. A two-way repeated-measures analysis of variance (ANOVA, shoe × time) was conducted to determine any differences in joint biomechanics. Main effects for shoe type were observed at the ankle, knee and hip joints during the stance phase. In particular, decreased range of motion (ROM) was observed using the bionic shoes for all three joints, and the joint moments also had significant changes except for the frontal plane of the hip. Main effects for time were also observed at the ankle, knee and hip joints. The ROM of the sagittal plane in the knee and hip decreased post-5 km running. The reduction of ankle dorsiflexion, hip flexion, hip adduction and hip internal rotation angles were observed post-5 km running, as well as the increase of ankle eversion and external rotation, knee adduction and internal rotation angles. The kinetics also exhibited significant differences between pre-5 km running and post-5 km running. The interaction effects only existed in the ROM of the hip sagittal plane, hip adduction angle and hip internal rotation angle. The results suggested that bionic shoes could be beneficial for strengthening muscle control, enhancing postural stability and proprioceptive ability. Footwear personalization could be a solution that benefits runners, reduces injury risk and improves running performance.

## 1. Introduction

Running as a type of physical activity is becoming increasingly popular throughout the world, and the number of people participating in running has increased rapidly over recent years. However, 19–79% of runners have suffered a running-related injury (RRI) each year [1,2], which is contradictory to the positive health benefits. The majority of these injuries result from overuse injuries [3], and the main injuries occur in the lower extremity, especially the knee and foot [1]. However, the complex associations between joint motion and running-related injury are poorly understood.

Running biomechanics has been used either to understand running injury etiology [4] or for investigating running economy [5]. Shoe design has been implicated in running-related injuries although the literature is inconclusive on their role related to running injury risk [6]. Despite continuous advancement and innovation in the design of running shoes, the incidence of running injuries has remained relatively stable over the past 40 years [1,7]. There is much debate in the literature as to whether footwear design influences running-related injuries, both positively and negatively [8]. To adapt to different runners, running styles and running conditions, abundant variations of running shoes have been developed. Many variations of shoe design exist; however, shoe sole constructions have been considered as one of the most important factors relating to running performance and the risk of running-related injury [6,9]. This is related to the importance of the foot soles interaction with the central nervous system in providing valuable tactile sensation feedback [10].

In recent years, shoes with unstable structural designs have become gradually more popular both as a therapeutic [11] and a functional aid [12]. In the process of walking or running, the changes in the sole structure will cause instability of the human body, forcing the body to continuously adjust its posture, strengthening and increasing the activation of muscles, and maintaining the balance of the body during exercise [13,14]. Unstable shoe construction can improve neuromuscular control and enhance muscular strength by reducing stability. The design concept of unstable shoes came from the concept of unstable training equipment, such as the development of Masai barefoot technology (MBT), which is derived from wobble board training [13,15]. Several studies have been conducted to scientifically investigate the effects of unstable shoes. Horsak et al. [16] found that MBT shoes can enhance muscle activity through the joint contraction of the agonists and antagonists in the lower limb joints. Stöggl et al. [13] suggested that unstable shoes have the function of improving performance and reducing the risk of injury. Taniguchi et al. [17] investigated the changes in joint movement, and also the kinetic changes, and this study showed that unstable shoes absorb shock in the early stance phase and generate a progressive force in the late stance phase of walking. Sobhani et al. [18] found that in healthy participants, rocker shoes can decrease ankle plantarflexion moment during the late stance phase of both running and walking. Boyer et al. [19] concluded that unstable shoes may provide potential therapeutic opportunities for running-related injuries at the ankle without causing a substantial risk to the knee or hip joints. However, Nigg et al. [20] found that there was no significant improvement in balance capacity after six weeks’ training with MBT shoes.

The mechanisms of running overuse injuries are multifactorial; however, muscle fatigue and weakness have been considered as primary factors [8,21]. The effects of fatigue on sensorimotor control of running have been studied in many different ways. Fatigue has been shown to affect muscle strength, proprioception, and cognitive function [22]. Studies [23,24] have shown that fatigue can lead to running biomechanics alterations after prolonged running. However, previous studies [18,19,25] only investigated the acute running biomechanics of unstable shoes. The biomechanical changes in the lower limbs observed during fatigue protocols and prolonged running (i.e., typical training runs) indicate that the acute comparisons of footwear conditions may not explain the runner’s adaptation to the preferred path of movement throughout a typical training run [26]. It is necessary to quantify the changes of unstable shoes during a typical prolonged training run, as this may be more related to overuse of running injuries.

Research on the changes in the biomechanics of the lower limbs for a prolonged running period, and how different footwear affect these changes are limited. Therefore, the present study aimed to investigate the influence of neutral and unstable footwear on lower extremity biomechanics following a 5 km treadmill running session in male recreational runners. The unstable shoes used in the present study were a combination of traditional unstable structure and bionic science, in which soles were individually designed according to the structure and morphology of the foot; therefore, the unstable shoes used in this study were also named bionic shoes. It is hypothesized that (1) joint angles and moments in the lower extremity will change post-5 km running either with a neutral or a bionic shoe, especially in the ankle joint, and (2) lower joint angles and moments would differ between neutral and bionic shoe conditions either pre-5 km or post-5 km running, especially of the angle range of motion (ROM).

## 2. Materials and Methods

### 2.1. Participants

Sixteen healthy male recreational heel strike runners (mean ± standard deviation (SD): age: 24.2 ± 1.7 years, height: 1.76 ± 0.04 m, mass: 72.0 ± 4.6 kg, BMI: 22.8 ± 0.7 kg/m^2^, shoe size: 42.3 ± 1.0 EUR) who ran a minimum of 20 km per week and had not run in an unstable shoe were recruited as experimental subjects for this study. Participants were recruited from sports clubs of Ningbo University and via social media. All participants were free from health problems and/or neuromuscular disorders and/or known gait impairments, and had no lower limb injuries in the previous six months. Prior to the experiment, all participants were provided with and signed the documented consent approved by the Institutional Review Board.

### 2.2. Experimental Procedures

**Figure 1:**
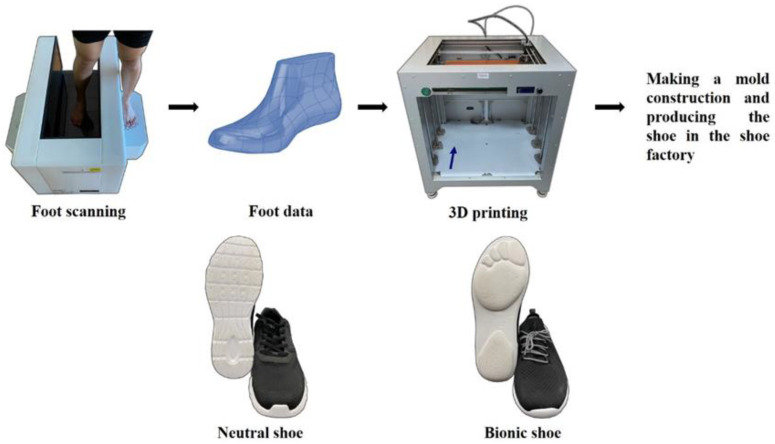
Illustration of making procedure of bionic shoes and neutral running shoes.

Participants completed 2 separate testing sessions in the biomechanics laboratory, with 7 to 10 days between testing sessions. For one of the sessions, participants wore bionic shoes, which contained two design parts. In initial part, a foot scanner machine (VAS 39, Ortho baltic, LITHUANIA) was used to scan foot profiles for each participant while the second part comprised the 3D print of the foot model (Dragon(L) 3D Printer, WINBO, Guangzhou, China) which was based on the scanning data. A mold construction for the shoe based on the results was developed in a Chinese factory (Ningbo Jiangbei Feibu Sports Goods Co., Ltd., Ningbo, China). For the other testing session, participants wore neutral running shoes with flat-soles (ART NO.11725599-7, ANTA) (Figure 1). The order of shoes worn in the running tests was randomly selected for the participants. The procedures for the experiment were the same for each running testing session.

**Figure 2:**
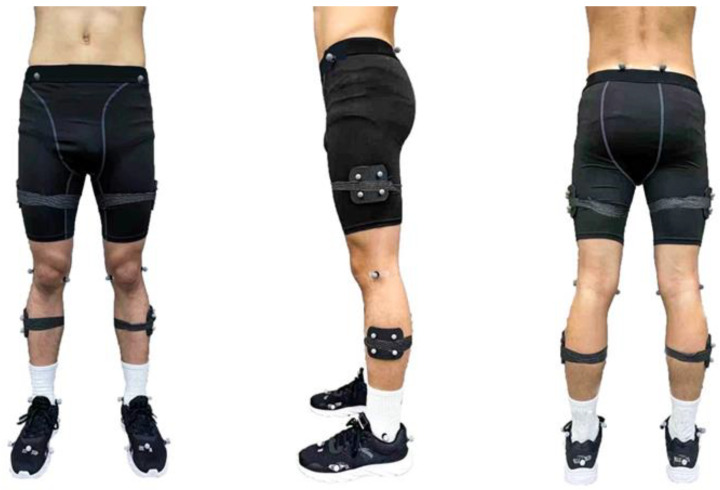
Illustration of retroreflective markers placement.

An eight-camera motion capture system (Vicon Metrics Ltd., Oxford, UK) was used to record running kinematic data at a frequency of 200 Hz, and an in-ground force plate (AMTI, Watertown, MA, USA) which was located in the middle of an overground runway recorded the ground reaction forces at 1000 Hz; 36 retroreflective markers were fixed to the lower limb of each runner to track movement [27], as outlined in Figure 2. Baseline data (pre-5 km running) were collected with the participant standing (static), and was then followed by running trials on the overground runway at their self-selected speed, which was considered as a “natural running pace”. This running speed was used for all running trials (pre- and post-5 km running). Timing gates were used to measure and control participants speed on the runway. Before testing, participants had a 10 min warm-up and time to familiarize themselves with procedures and instrumentation. The participants completed 5 successful running trials on their dominant leg (defined as the preferred leg when kicking a ball and all the participants’ right legs were the dominant limb) striking the force plate.

Following the baseline running test, participants provided their average speed for a 5km run (in minutes per mile), and then ran 5 km on the treadmill at their self-selected speeds (which were recorded at 3.09 ± 0.16 m/s, and in the range of 10–12 km/h). Participants were given 2 min to warm up on the treadmill, once the treadmill speed was set, participants ran at that speed for 5 km. During the 5 km running session, all retroreflective markers remained on the participants. The post 5 km test started within 2 min of finishing the treadmill run using the same test protocols as the baseline test.

### 2.3. Data Analysis

The stance phase of running inclusive of the right heel strike to toe-off was analyzed in this study. A customized function in Visual 3D (c-motion Inc., Germantown, MD, USA) was applied to process and quantify kinematic and kinetic variables in the stance phase of the ankle, knee and hip joints using C3D files generated by Vicon Nexus Software. The data of kinematics and kinetics were filtered by 10 Hz and 20 Hz fourth-order zero-phase low pass Butterworth filter for the de-noising process of marker trajectories [28]. The standard inverse dynamic method was used to calculate the internal joint moments and joint powers. The joint kinetic data were normalized for the participant’s body mass. Joint kinematic and kinetic data were time normalized to the stance phase (101 data points per stance phase) by Matlab version 2019b (The Math Works, Natick, MA, USA).

### 2.4. Statistical Analysis

A two-way repeated-measures analysis of variance (ANOVA) was used (shoe × time) to test for group differences (bionic shoe vs. neutral shoe) and to evaluate if there were any group by 5 km run interaction. Firstly, ANOVA assumptions (normality and homogeneity of residuals) were examined. When assumptions were met, a two-way repeated-measures ANOVA was used to evaluate the main effects of ‘shoe’ and ‘time’ factors, and the interaction of the two factors. When the assumptions of ANOVA were not satisfied, a permutation procedure was performed. Alpha level was set to α = 0.05. While the interaction effect was significant (*p* < 0.05), post-hoc pairwise comparisons with a Bonferroni correction (α = *p*/6 = 0.008) were applied. The statistical calculations were carried out using SPSS version 25.0 software (IBM, Armonk, NY, USA).

Due to the one-dimensional time varying characteristics of joint kinematics and joint kinetics [23]. Two-way repeated-measures ANOVAs (shoe × time) were applied by using one-dimensional statistical parametric mapping (SPM1D) to evaluate the main effects of ‘shoe’ and ‘time’ factors and their interaction of two factors and to compare mean joint angle and joint moment waveforms over the stance phase. SPM1D relies on random vector field theory to account for data variability. The statistical analyses were completed in Matlab version 2019b (Mathworks Inc.), and the significance level was set at *p* < 0.05.

## 3. Results

### 3.1. Effects of the Shoe Condition

**Figure 3:**
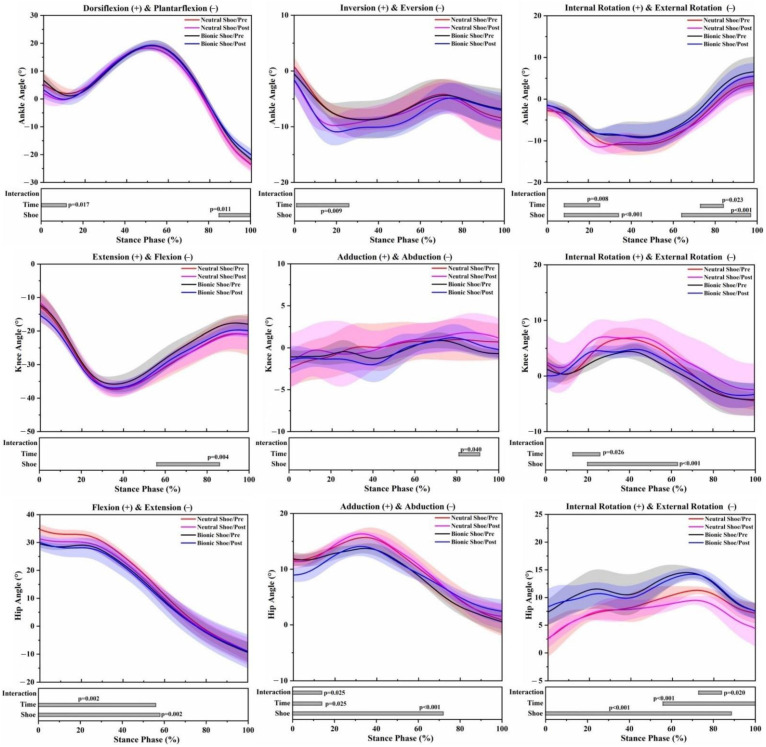
Lower limb joint angle waveforms of mean and standard deviation over the stance phase of 4 running conditions. Significant main effects of the shoe, time and interaction (*p* < 0.05) are highlighted (grey horizontal bars at the bottom of the figure) during corresponding periods from SPM1d analyses.

**Figure 4:**
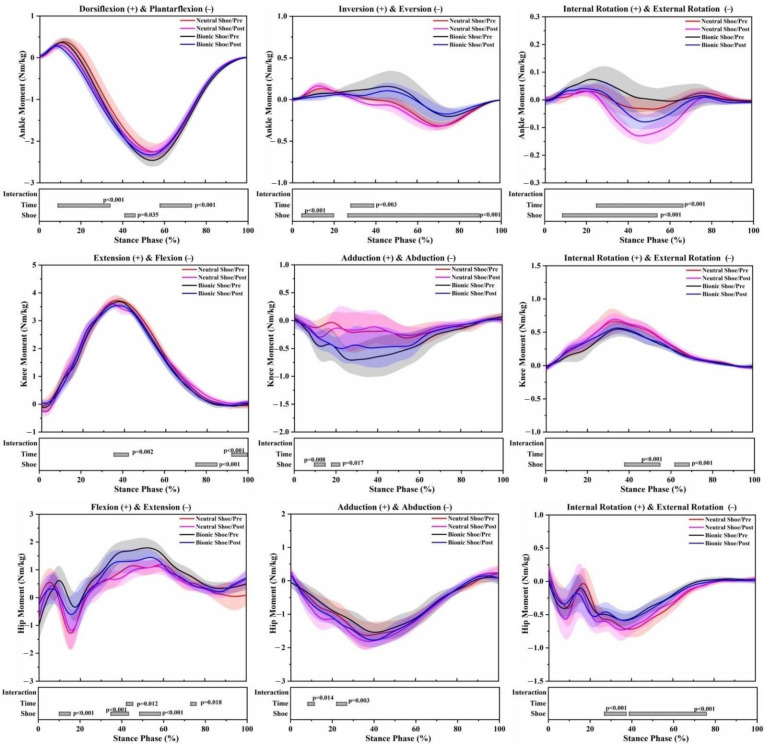
Lower limb joint moment waveforms of mean and standard deviation over the stance phase of 4 running conditions. Significant main effects of the shoe, time and interaction (*p* < 0.05) are highlighted (grey horizontal bars at the bottom of the figure) during corresponding periods from SPM1d analyses.

In both pre-5 km running and post-5 km running, the angle range of motion (ROM) of the bionic shoe showed significant decreases in the ankle sagittal plane (F = 6.813; *p* = 0.020), knee sagittal plane (F = 8.823; *p* = 0.010), knee horizontal plane (F = 13.675; *p* = 0.002), hip sagittal plane (F = 14.138; *p* = 0.002) and knee frontal plane (F = 50.948; *p* < 0.001) compared to the neutral shoe (Table 1). At the ankle joint of the bionic shoe, the plantarflexion angle decreased by 85–100% (*p* = 0.011), external rotation angles increased by 8–34% (*p* < 0.001) and 64–97% (*p* < 0.001) (Figure 3). Increased plantarflexion moment was observed across the stance phase by 42–46% (*p* = 0.035), inversion moment decreased by 5–20% (*p* < 0.001) and increased by 27–90% (*p* < 0.001). Decreased external rotation moment was observed at 9–54% (*p* < 0.001) (Figure 4). At the knee joint of the bionic shoe, flexion angles decreased by 57–86% (*p* = 0.004), internal rotation angle decreased by 20–63% (*p* < 0.001) (Figure 3). Decreased extension moments were observed at 75–85% (*p* < 0.001), abduction moment increased by 10–15% (*p* = 0.004) and 18–22% (*p* = 0.017), internal rotation moment decreased by 38–55% (*p* < 0.001) and 62–69% (*p* < 0.001) (Figure 4). At the hip joint of the bionic shoe, flexion angle decreased by 0–58% (*p* = 0.002), adduction angle decreased by 0–72% (*p* < 0.001), and internal rotation angle increased by 0–87% (*p* < 0.001) (Figure 3). Increased flexion moment was observed at 10–15% (*p* < 0.001), 35–43% (*p* < 0.001) and 49–59% (*p* < 0.001). External rotation moment decreased by 27–37% (*p* < 0.001) and 38–76% (*p* < 0.001) (Figure 4).

### 3.2. Effects of the 5 km Run (Time)

The angle ROM of the post-5 km running showed significant decreases in the knee sagittal plane (F = 6.136; *p* = 0.026) and hip sagittal plane (F = 4.944; *p* = 0.042) (Table 1), regardless of shoe condition. At the ankle joint of post-5 km running, dorsiflexion angles decreased by 0–12% (*p* = 0.017), eversion angle increased by 1–26% (*p* = 0.009), external rotation angle increased by 8–25% (*p* = 0.008) and 73–85% (*p* = 0.023) (Figure 3). Increased plantarflexion moment was observed at 8–34% (*p* < 0.001) and 58–73% (*p* < 0.001), inversion moment decreased by 28–39% (*p* = 0.003), external rotation moment increased by 25–66% (*p* < 0.001) (Figure 4). At the knee joint of post-5 km running, the adduction angle increased by 81–91% (*p* = 0.040), internal rotation increased by 13–27% (*p* = 0.026) (Figure 3). Extension moment decreased by 36–43% (*p* = 0.002) and increased by 92–100% (*p* < 0.001) (Figure 4). At the hip joint of post-5 km running, flexion angle decreased by 0–56% (*p* = 0.002), adduction angle decreased by 0–14% (*p* = 0.025), internal rotation angle decreased by 56–100% (*p* < 0.001) (Figure 3). Flexion moment decreased by 42–45% (*p* = 0.012) and 73–76% (*p* = 0.018), abduction moment increased by 8–11% (*p* = 0.014) and 22–27% (*p* = 0.003) (Figure 4).

### 3.3. Interaction Effects

The interaction between the shoe condition and 5 km run induced a significant effect on angle ROM of hip sagittal plane (F = 13.252; *p* = 0.002) (Table 1), hip adduction angle (*p* = 0.025) and hip internal rotation angle (*p* = 0.020) (Figure 3).

## 4. Discussion

The purpose of the present study was to investigate the effects of bionic shoes on lower extremity running biomechanics before and after a 5 km run compared with a neutral running shoe. Although there have been several studies investigating the sports biomechanics of unstable running shoes, we believe this is the first scientific research to make a comparison in the literature. The results support our hypothesis, showing joint angles and moments in the lower extremity changed in post-5 km running either with a neutral or a bionic shoe, and lower extremity biomechanics were different between the neutral and bionic shoe conditions either pre-5 km or post-5 km running.

Footwear design may influence the human motor control system during running [29]. Previous studies [15,18] have shown that unstable shoes can cause changes in the kinematics of the lower limbs. Similar results were found in our study. ROM of all three joints showed a significant decrease for bionic shoes compared to neutral running shoes. The decrease of the joint angle ROM usually indicates a strengthening in muscle control, which may increase the activation of the ankle muscles [25]. The reduction of ankle plantarflexion was observed in bionic shoes. A possible explanation for this is that, due to the unstable outsole construction, the rearfoot and forefoot are heightened and the area between the ankle joint and ground is enlarged to prevent stumbling while running, thereby providing a smaller plantarflexion to adjust for the change [30]. The flexion angle of the hip and knee also showed degrees of decrease, which were consistent with previous studies [30,31,32]. Due to the instability of the lower limbs in the stance phase, the body adjusts to a more stable running style by reducing the flexion angle of the hip and knee joints. The joint angle rotation changed greatly in all three lower limb joints using the bionic shoes regardless of the 5 km run. The results showed a reduction in the external rotation angle of the ankle, a reduction in internal rotation of the knee, but an increase in internal rotation of the hip. In order to maintain stability, from the perspective of neurophysiology and anatomy, the posture control system could expand the amplitude of the lateral rotation of the knee joint and strengthen the movement of the internal rotation of the hip joint [15,33]. The abduction angle of the hip with bionic shoes was reduced significantly, which may be the result of the neuromuscular system, sensorimotor system and proprioception adjusting the range of motion of the lower limbs to a relatively safe range to avoid injury. Bionic shoes induced less eversion and external rotation moments of the ankle than neutral running shoes. Previous studies [13,15] suggested that the unstable element of the outsole could strengthen muscle control. This may reduce ankle joint activity in the frontal plane and horizontal plane during the stance phase, thereby shortening the force arm of ankle eversion and external rotation. Due to the significant changes in the ankle joint, the knee joint and hip joint will also be followed by compensatory alterations, less net knee and hip moments may avoid an early onset on fatigue during prolonged running sessions [34]. The bionic soles designed in this study may be used to increase neuromuscular strength, and to a certain extent enhance stability and proprioception.

Fatigue is an important factor affecting lower limb biomechanics during running [22,23]. After 5 km treadmill running, the ROM of the knee sagittal plane showed a decreasing trend, as well as the sagittal plane of the hip joint during the stance phase of running. Previous studies [35,36] found that the joint ROM increased in a fatigued state, however, our statistical results are not consistent with this observation. This may be due to the large inter-individual variability in the kinematics of running. Dorsiflexion angles decreased during post-5 km running for the impact phase of the stance, which has been shown in previous studies and has been cited as the result of foot dorsiflexor fatigue [37]. The fatigue of the dorsiflexor may increase the range of amplitude swings, making the ankle joint more likely to be injured [38]. Ankle eversion and external rotation angles increased during post-5 km running, and this may suggest that posture control was affected after a prolonged running session. The increase of knee abduction and internal rotation angles was observed in post-5 km running, which may be undesirable for runners as increased frontal and horizontal plane knee and ankle excursion has been recognized as a cause of knee pain [39,40]. The hip joint was followed by compensatory changes due to the significant changes of angles in the ankle and knee joints. The joint kinetic variables following a 5 km run were consistent with a recent study by Mei et al. [23] of the lower extremity changes after long-distance running. The increase in ankle plantarflexion, eversion and external rotation moments were observed during post-5 km running. In the event of fatigue, that is a challenge to the habitual motion pathways, extra muscle forces are required to create the ankle joint moments necessary to keep the ankle in the habitual path [41,42]. Knee extension moments significantly decreased in mid stance following the 5 km run, which may be associated with the weak extensor muscles reported in recreational runners [43]. We found decreased extension moments and increased abduction moments of the hip joint, and it has been reported that hip flexor and abductor muscle strength is reduced in overuse injuries of recreational runners [23,43]. The reduction in muscle strength leads to an imbalance in hip joint moments and the net result is decreased extension and increased abduction moments.

It has recently been proposed that the neuromuscular system regulates motion mechanics by minimizing the load on biological structures when following the preferred or habitual path of motion [6,41]. In the presence of constraints, such as inappropriate footwear/insoles or fatigue states, the retention of these motion paths is challenged, and the biomechanics of the lower extremities are modified in a necessary way to keep the joints on their preferred or habitual path. Further research should explore our understanding of whether footwear should be designed to maintain the initial habitual path of motion or whether footwear should provide support when muscles may experience fatigue and the habitual motion path has changed [26]. A compromise must be found between running performance optimization and running protection in shoe design.

There are several limitations in the current study. Firstly, we only investigated the stance phase of running, as it has been reported that the running stance phase is closely associated with running-related injuries [44,45]. However, footwear and prolonged running could considerably influence lower limb biomechanics during the swing phase of gait. Secondly, the outsoles of the bionic shoes used in this investigation were manufactured in relation to the structure and morphology of the foot. The joint biomechanics may be different when compared with other commercially available unstable shoes, such as MBT shoes. Thirdly, differences in shoe uppers and materials between bionic shoes neutral shoes may cause additional interference in running biomechanics, as well as the shoe weight induced by the shoe size and material used. Since the shoes were not matched for weight in this study, and we know that the increased weight of shoes is negatively influential on running economy, it would be worthwhile performing a similar study in future to see how bionic shoes would affect running biomechanics after correcting for shoe mass. Finally, the participants of this study were all healthy male recreational runners and consequently, the findings of our study may not apply to females and injured runners. Those factors should be considered in future research.

## 5. Conclusions

This study showed that bionic shoes altered running biomechanics in the lower extremity during the stance phase. The results provided practical evidence for footwear design suggesting that the combination of traditional unstable elements and bionic science could be beneficial for strengthening muscle control, enhancing postural stability and proprioceptive ability. Footwear personalization could be a solution that benefits runners to reduce injury risk and improve running performance. The findings of the present study may contribute to our understanding of the effects of footwear and prolonged running, and provides valuable information on preventing running-related injuries. Future work should examine the effects of runners running in bionic shoes over a range of longer time periods of one to eight weeks.

## Figures and Tables

**Table 1:** Mean (SD) of angle range of motion (ROM) of the stance phase for the four experimental conditions.

Joint	Variables	Neutral Shoe/Pre	Neutral Shoe/Post	Bionic Shoe/Pre	Bionic Shoe/Post	Main Effect Shoe	Main Effect Time	Interaction Effect
Ankle	Sagittal ROM (°)	42.90 (2.20)	42.30 (2.46)	41.10 (3.82)	39.58 (3.63)	**F = 6.813; *p* = 0.020**	F = 1.813; *p* = 0.198	F = 0.602; *p* = 0.450
	Frontal ROM (°)	10.55 (3.53)	10.04 (2.80)	9.00 (3.04)	9.87 (1.23)	F = 4.490; *p* = 0.051	F = 0.049; *p* = 0.827	F = 0.696; *p* = 0.417
	Horizontal ROM (°)	15.30 (2.78)	15.22 (2.16)	16.58 (4.34)	15.87 (4.16)	F = 0.843; *p* = 0.373	F = 0.654; *p* = 0.431	F = 0.282; *p* = 0.603
Knee	Sagittal ROM (°)	26.86 (3.42)	25.78 (3.53)	23.90 (4.53)	21.77 (3.39)	**F = 8.823; *p* = 0.010**	**F = 6.136; *p* = 0.026**	F = 0.570; *p* = 0.462
	Frontal ROM (°)	4.66 (1.34)	4.36 (1.59)	3.30 (0.97)	4.68 (2.40)	F = 0.684; *p* = 0.421	F = 3.322; *p* = 0.088	F = 3.120; *p* = 0.030
	Horizontal ROM (°)	11.74 (1.09)	10.59 (2.34)	9.70 (2.03)	9.18 (1.50)	**F = 13.675; *p* = 0.002**	F = 4.675; *p* = 0.057	F = 0.333; *p* = 0.572
Hip	Sagittal ROM (°)	43.64 (3.99)	40.44 (4.35)	39.17 (3.32)	39.16 (5.55)	**F = 14.138; *p* = 0.002**	**F = 4.944; *p* = 0.042**	**F = 13.252; *p* = 0.002**
	Frontal ROM (°)	14.93 (1.84)	14.95 (2.20)	13.50 (2.39)	11.69 (0.87)	**F = 50.948; *p* < 0.001**	F = 4.180; *p* = 0.059	F = 5.986; *p* = 0.027
	Horizontal ROM (°)	9.41 (2.99)	8.87 (2.45)	9.25 (1.55)	7.84 (1.69)	F = 0.911; *p* = 0.355	F = 3.237; *p* = 0.092	F = 0.426; *p* = 0.524

Note: Statistical significance was set to *p* < 0.05. The significant differences in interaction effect were based on the results of Bonferroni post hoc tests (α = 0.008). The bold represented significant differences.

## Data Availability

The data that support the findings of this study are available on reasonable request from the corresponding author. The data are not publicly available due to privacy or ethical restrictions.
